# The Role of Human Viral Entry Receptor Mouse Models in Advancing Antiviral Antibodies and Vaccines

**DOI:** 10.3390/vaccines14070614

**Published:** 2026-07-14

**Authors:** Na Zuo, Xin Zheng, Rameez Ishaq, Deshan Ren, Ao Hu

**Affiliations:** 1 Department of Obstetrics and Gynecology, Union Hospital, Tongji Medical College, Huazhong University of Science and Technology, Wuhan 430022, China; 2 Department of Biotechnology, Balochistan University of Information Technology, Engineering and Management Sciences, Quetta 87300, Pakistan; 3 National Resource Center for Mutant Mice and MOE Key Laboratory of Model Animal for Disease Study, Model Animal Research Center, Medical School of Nanjing University, Nanjing 210061, China; 4 Wuhan Institute of Virology, Chinese Academy of Sciences, Wuhan 430071, China

**Keywords:** receptor-humanized mouse models, virus, vaccine development, CRISPR-Cas9, neutralizing antibodies, humanized mice

## Abstract

Human viral entry receptor mouse models exist to overcome a fundamental experimental barrier: many clinically important viruses bind their human entry factors far more efficiently than the corresponding murine orthologs, leaving conventional mice unable to support authentic infection, physiological tissue tropism, or meaningful countermeasure evaluation. This review is organized around the receptor-humanization concept rather than around a single coronavirus model. Engineering strategies compared here include random transgenesis, endogenous-locus knock-in, minimal receptor-interface humanization, conditional and inducible expression, and transient vector-mediated delivery. Receptor systems covered span human angiotensin-converting enzyme 2 (hACE2)-dependent sarbecoviruses, human dipeptidyl peptidase 4 (hDPP4)-dependent Middle East respiratory syndrome coronavirus (MERS-CoV), human cluster of differentiation 4/human C-C chemokine receptor type 5 (hCD4/hCCR5)-dependentt human immunodeficiency virus type 1 (HIV-1), adenovirus receptor models, human intercellular adhesion molecule 1 (hICAM-1) rhinovirus systems, hepatitis C virus (HCV), hepatitis B virus (HBV), and hepatitis D virus (HDV) entry-factor models, measles receptor models, poliovirus receptor/CD155 (PVR/CD155) models, human scavenger receptor class B member 2 (hSCARB2) enterovirus systems, and human transferrin receptor 1 (hTfR1) arenavirus models. We then discuss how these platforms support antibody evaluation, Fc-effector analysis, vaccine protection, variant benchmarking, and safety assessment. These models yield the most reliable data when the experimental question is explicitly entry-dependent and when receptor expression level, anatomical distribution, pathology window, and immune context have all been independently validated. They are least informative when receptor expression is non-physiological, when disease readouts are driven by promoter artifacts, or when post-entry species barriers remain the dominant bottleneck. A validation-centered framework is therefore proposed to guide the selection of each model for the specific antiviral antibody or vaccine question it can legitimately answer.

## 1. Introduction: From Species Barriers to Receptor-Humanized Infection Models

Mouse models humanized for viral entry receptors represent one class within the broader family of genetically humanized infection platforms. Their greatest value arises when receptor engagement itself constitutes the dominant species barrier. The central issue is not simply that mice are experimentally convenient, but that many human viruses recognize their cognate human receptors far more efficiently than the corresponding murine orthologs. Previous reviews have therefore emphasized that receptor-humanized mice are best used as hypothesis-driven platforms for restoring viral entry competence rather than as generic replicas of human disease [1,2,3,4,5]. This principle applies across otherwise unrelated virus families, including human angiotensin-converting enzyme 2 (hACE2) and human dipeptidyl peptidase 4 (hDPP4) for coronaviruses, human intercellular adhesion molecule 1 (hICAM-1) for major-group rhinoviruses, human sodium taurocholate cotransporting polypeptide (hNTCP) for hepatitis B and D viruses, poliovirus receptor/CD155 (PVR/CD155) for poliovirus, human scavenger receptor class B member 2 (hSCARB2) for enterovirus A71 (EV-A71), human transferrin receptor 1 (hTfR1) for New World arenaviruses, and human cluster of differentiation 4 (hCD4), human C-C chemokine receptor type 5 (hCCR5), or liver-specific entry factors for viruses that encounter additional post-entry restrictions [1,3,4,5].

Receptor-humanized mice are most informative when the experimental question is explicitly receptor dependent. For example, when the objective is to determine whether a neutralizing antibody blocks a defined human receptor-dependent entry event or whether vaccine-induced immunity protects against a virus whose host range is governed by that receptor, these models provide a rigorous in vivo bridge between cell-culture assays and large-animal or clinical studies. By contrast, receptor humanization alone is generally insufficient for investigating chronic infection, adaptive immune selection, antibody maturation, human leukocyte antigen (HLA)-restricted T-cell immunodominance, or human fragment crystallizable (Fc) receptor biology. Murine immune signaling, Fc receptor networks, stromal architecture, cytokine programs, and tissue-repair pathways remain distinct from their human counterparts; therefore, model selection must account for immune and tissue context in addition to viral entry [6,7].

In this narrative review, we synthesize recent advances in the development, validation, and application of mouse models humanized for viral entry receptors. We examine how distinct engineering strategies, including random transgenesis, endogenous-locus knock-in, minimal receptor-interface humanization, conditional or inducible expression, and transient vector-mediated delivery, shape viral susceptibility, tissue tropism, disease phenotypes, and the interpretation of antiviral studies. This review has three main objectives: to compare model designs across representative viral receptor systems; to summarize their applications in evaluating antiviral antibodies, vaccine efficacy, Fc-dependent functions, variant breadth, and safety; and to define the biological and technical limitations that constrain their translational relevance. Although hACE2- and hDPP4-based coronavirus models receive particular emphasis because they represent the most extensively developed evidence base, representative models for other clinically important viruses are also discussed to provide a broader cross-viral perspective.

**Figure 1:**
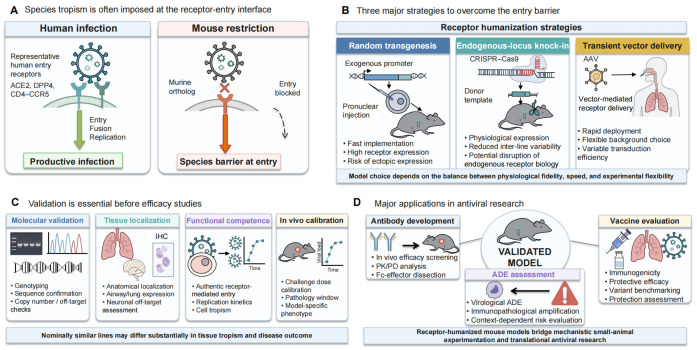
Engineering, validation, and applications of receptor-humanized mouse models. (**A**) Many clinically important human viruses fail to infect standard laboratory mice because the corresponding murine receptor orthologs do not support efficient viral attachment or entry. In permissive human cells, receptor engagement enables attachment, entry, and productive infection, whereas inefficient engagement of the murine ortholog creates a species barrier at the entry stage. (**B**) This entry barrier can be overcome through three major receptor-humanization strategies: random transgenesis, clustered regularly interspaced short palindromic repeats/CRISPR-associated protein 9 (CRISPR/Cas9)-mediated endogenous-locus knock-in, and transient adeno-associated virus (AAV)-mediated receptor delivery. These approaches differ in implementation speed, physiological fidelity, experimental flexibility, and susceptibility to artifacts such as ectopic expression or variable transduction efficiency. (**C**) Rigorous validation is required before efficacy studies, including molecular confirmation of the engineered allele, anatomical characterization of receptor expression, immunohistochemistry (IHC) where appropriate, functional demonstration of authentic receptor-dependent entry, and in vivo calibration of the challenge dose and pathology window. (**D**) Once established and validated, receptor-humanized mouse models support multiple applications in antiviral research, including in vivo antibody screening, pharmacokinetic/pharmacodynamic (PK/PD) analysis, antibody-dependent enhancement (ADE) assessment, and vaccine evaluation based on immunogenicity, protective efficacy, immune readouts, and variant benchmarking.

The engineering strategy chosen at the outset constrains every downstream interpretation. Random transgenesis remains fast and experimentally accessible, but copy number, insertion site, and promoter choice can create expression patterns that are useful for robust challenge yet misleading for tissue tropism. Endogenous-locus knock-in preserves more physiological regulation but may yield a narrower disease window. Conditional, inducible, and precision-editing strategies add temporal or cell-type control, and prime editing illustrates how small receptor-interface changes may eventually allow entry competence without replacing an entire gene [8,9,10,11,12,13]. This review therefore emphasizes a validation-centered framework: first define the receptor-dependent question, then choose the model architecture, and only then interpret antibody, vaccine, or pathogenesis readouts within the limits of that architecture. As shown in Figure 1, this decision path links model-engineering choices to the downstream antibody, vaccine, and variant-benchmarking questions that each platform can legitimately support.

## 2. Building Receptor-Humanized Models: Design Choices That Shape Interpretation

The first practical decision is whether susceptibility should be maximized or physiological fidelity should be prioritized. Random transgenesis can rapidly produce a line in which a human receptor is expressed at high levels, often under an epithelial or broadly active promoter. This can be valuable when the goal is to establish a reproducible disease window for screening many antibody or vaccine candidates. The limitation is that the construct, the integration site, and the founder line become part of the phenotype. Two animals may carry the same receptor sequence but differ substantially in receptor abundance, tissue distribution, and disease severity because random integration places the transgene under different chromatin and regulatory influences. Classic transgenic technology therefore remains powerful, but it should be interpreted as a line-specific experimental system rather than as a direct physiological substitute for the human receptor locus [8,9,10].

Targeted knock-in and minimal interface humanization address a different problem. By placing the human receptor sequence, or selected human-like residues, into a defined genomic context, these approaches reduce founder-line variability and align expression more closely with endogenous regulatory logic. They are particularly attractive when tissue tropism, cell-type restriction, or chronic tissue injury is part of the question. The trade-off is that physiological receptor distribution may produce a milder phenotype, requiring more careful endpoint selection and larger cohorts [8]. Conditional and inducible systems introduce a further layer of experimental precision: tetracycline-responsive designs allow receptor expression to be tuned over time, while Cre-loxP strategies confine it to selected cell types or developmental windows [11,12]. Prime editing and related precision-editing approaches are conceptually important because receptor usage is often determined by a small number of interface residues; in such cases, precise nucleotide changes may create entry competence while leaving the remainder of the receptor and its regulatory architecture intact [13].

Validation is a core experimental result, not a technical appendix. At minimum, a receptor-humanized model must be characterized at the allele, RNA, protein, anatomical, and functional levels. Molecular confirmation verifies that the intended receptor sequence is present and that no obvious rearrangement has occurred; tissue-level validation asks whether the receptor is found in the relevant cell types; and functional validation asks whether infection is actually receptor dependent. This is especially important for sarbecoviruses because severe acute respiratory syndrome coronavirus (SARS-CoV) and severe acute respiratory syndrome coronavirus 2 (SARS-CoV-2) both use ACE2 but differ in receptor-binding details, protease usage, and experimental disease phenotype. Structural and entry studies therefore support the principle that SARS-CoV data cannot automatically substitute for SARS-CoV-2 data, or vice versa, when the claim concerns a pathogen-specific entry event [14,15,16,17].

A useful model-selection rule is to align the model with the narrowest claim the experiment needs to support. When the intended claim is entry blockade, high and reproducible receptor-dependent susceptibility may be acceptable even if expression is not fully physiological. When the claim is tissue tropism, disease mechanism, or safety, promoter and locus become central. When the claim concerns vaccine immunogenicity or antibody effector function, receptor humanization may need to be paired with immune-system or Fc-receptor humanization. This is why a single ranking of receptor-humanized models is misleading: the better model is the one whose engineered feature matches the biological claim and whose remaining limitations have been measured rather than assumed [6,7,8,9,10,11,12,13].

The same validation logic also explains why apparently similar hACE2 models can answer different questions. Keratin-18 promoter-driven hACE2 (K18-hACE2) mice, other transgenic hACE2 lines, endogenous hACE2 knock-in mice, adeno-associated virus (AAV)-mediated hACE2 delivery systems, and mouse-adapted SARS-CoV-2 all solve the susceptibility problem in different ways. A strong transgenic model may be excellent for rapid countermeasure ranking but may also carry promoter-driven tissue distribution that complicates interpretation. A knock-in model may better preserve receptor regulation but can produce a subtler disease window. AAV-hACE2 delivery can be deployed quickly in multiple mouse backgrounds, whereas mouse-adapted viruses bypass receptor humanization altogether and therefore answer a different question. For this reason, model reports should specify receptor locus or promoter, receptor distribution, genetic background, viral strain, challenge window, and the endpoint being used to judge protection or disease [18,19,20,21,22,23,24,25,26,27,28].

## 3. Coronavirus Entry-Receptor Models: Lessons from hACE2 and hDPP4

### 3.1. hACE2 Models: Transgenic, Knock-In, and Vector-Based Solutions

hACE2 models are the most familiar receptor-humanized systems, and they remain the clearest illustration of why model construction determines interpretation. SARS-CoV and SARS-CoV-2 both use ACE2, but the supporting evidence for receptor engagement, spike structure, entry cofactors, and tissue phenotype must be pathogen specific [14,15,16,17]. The original K18-hACE2 line was developed for SARS-CoV studies and later became a widely used SARS-CoV-2 model because it provides a strong, reproducible disease window [18,19,20,21,22]. That strength is also its principal caveat: the keratin-18 promoter distributes receptor expression beyond the narrowest physiological range, and severe disease can reflect the combined influence of viral replication, receptor abundance, tissue distribution, and host inflammatory response working in concert. Such models are therefore highly useful for protection experiments but should not be described as uniformly physiological models of human coronavirus disease 2019 (COVID-19).

Endogenous-locus hACE2 knock-in and related humanized *Ace2* systems arose from the need to separate receptor usage from promoter artifact. Knock-in mice support SARS-CoV-2 infection in respiratory and intestinal tissues while aligning receptor expression more closely with endogenous regulation [23,24,25]. More recent hACE2 and conditional hACE2 designs further expand the toolkit by allowing investigators to tune susceptibility, compare tissue contributions, or restrict receptor expression to defined contexts [24,25,26]. These models usually provide cleaner receptor biology but may generate lower viral titers or less dramatic clinical disease than K18-hACE2 mice. That trade-off is not a weakness; it simply means that knock-in models are better suited to questions about tissue-relevant entry and pathogenesis, whereas high-susceptibility transgenic models may be better suited to screening when a robust effect size is needed.

A further issue in hACE2 model interpretation is that disease severity is not the same as receptor fidelity. Severe weight loss, high lung viral load, and lethality can be advantageous for ranking countermeasures, but they can also compress the window in which mechanistic differences are visible. Conversely, a milder knock-in phenotype may look less dramatic but may be preferable when the question is whether infection occurs in a tissue distribution closer to endogenous ACE2 biology. The model should therefore be selected before the experiment based on whether the desired readout is sensitivity, physiological distribution, tissue pathology, or therapeutic discrimination [18,19,20,21,22,23,24,25,26,27,28].

Vector-mediated hACE2 delivery and mouse-adapted SARS-CoV-2 are useful complementary strategies. AAV-hACE2 delivery can rapidly establish receptor-dependent susceptibility without maintaining a dedicated colony, making it valuable when multiple genetic backgrounds or immune perturbations need to be tested [27]. Mouse-adapted SARS-CoV-2 creates a tractable challenge model without changing the host receptor, which is useful for countermeasure studies but no longer tests the same receptor-humanization premise [28]. These systems should therefore be described with precision: they are not interchangeable with endogenous hACE2 knock-in models, but they are important tools when the experimental question prioritizes speed, strain flexibility, or a defined disease window.

### 3.2. hDPP4 Models: MERS-CoV as a Second Coronavirus Test Case

Middle East respiratory syndrome coronavirus (MERS-CoV) broadens the coronavirus discussion because its receptor is DPP4/CD26 rather than ACE2. DPP4 was identified as the functional receptor for MERS-CoV when the virus first emerged in 2012, and species differences in the DPP4 sequence explain why conventional mice are largely refractory to infection by wild-type MERS-CoV [29,30]. This receptor barrier led to a family of human DPP4 (hDPP4)-based systems, including rapid receptor-expression models, transgenic mice, knock-in mice, and models that combine hDPP4 expression with virus adaptation [31,32,33,34,35,36,37]. Together they show that a receptor-humanized review cannot be restricted to hACE2: even within coronaviruses, receptor identity, tissue distribution, and disease phenotype differ enough that model-specific discussion is essential.

MERS-CoV models also show why receptor-humanized mice should be discussed as families of platforms rather than as one named animal. Rapid expression systems were useful for establishing susceptibility and early countermeasure testing, while random transgenic and knock-in approaches allowed more stable comparison of pathology and protection. Models that combine hDPP4 expression with viral adaptation further increase disease robustness but introduce another layer of interpretation: protection may reflect both human-receptor entry and properties selected during adaptation. This does not invalidate the model, but it means that antibody, vaccine, and pathogenesis studies must state the exact host-virus combination being tested [31,32,33,34,35,36,37].

The hDPP4 literature is also valuable because it demonstrates how different engineering solutions can be selected for different endpoints. Some hDPP4 transgenic lines produce severe multi-organ disease and a strong intervention window, whereas knock-in and mouse-adapted systems can provide different balances of lung pathology, immune competence, and experimental reproducibility [31,32,33,34,35,36,37]. For antibody and vaccine studies, these models allow spike-directed interventions to be tested in an entry-relevant in vivo setting. However, protection data should always be reported with the exact platform used, because transgenic, knock-in, and adapted-virus approaches can differ in disease kinetics, tissue involvement, and the magnitude of protection observed [31,32,33,34,35,36,37].

## 4. Beyond Coronaviruses: What Other Receptor-Humanized Models Teach

### 4.1. HIV-1, Adenovirus, and Rhinovirus: Entry Competence Versus Biological Context

HIV-1 is an instructive limit case because receptor humanization can open the door to entry while leaving much of the biologically relevant disease process outside the reach of receptor expression alone. Human CD4 and CCR5 can confer entry competence in engineered mice, and models incorporating human cyclin T1 address additional transcriptional restrictions [38,39]. Nevertheless, questions about HIV-1 persistence, latency, mucosal transmission, antibody evolution, and T-cell exhaustion generally require broader human immune-system reconstitution [40,41]. HIV-1 therefore illustrates one of the review’s central organizing principles: a receptor-humanized model can be exactly the right tool for an entry question and entirely the wrong tool for a chronic immunological one. The appropriate model is defined not by the virus name alone but by the step in the viral life cycle or immune response being interrogated [3,6,38,39,40,41].

Adenovirus receptor models raise a related but distinct issue: receptor usage can shape vector transduction even when the goal is antigen delivery rather than natural viral disease. Human coxsackievirus and adenovirus receptor (hCAR) expression enabled adenoviral-mediated delivery into naïve T cells, and human CD46 (hCD46)-transgenic mice supported studies of species-B adenoviral vectors that use CD46 as a cellular receptor [42,43,44]. These systems are relevant to vaccine research because adenoviral vectors are widely used as antigen-delivery platforms, but interpretation must distinguish vector tropism from productive infection. In this setting, the receptor-humanized model helps define which cells receive the vector and how that distribution affects immune readouts, not whether the full natural history of adenovirus disease has been reproduced [42,43,44].

Rhinovirus hICAM-1 systems show the therapeutic value of receptor-humanized models in a more focused airway context. Major-group rhinoviruses use ICAM-1 for entry, and human ICAM-1 transgenic mice have been used to model rhinovirus-induced airway disease and exacerbation of allergic inflammation [45]. Importantly, an anti-human ICAM-1 antibody reduced rhinovirus-driven exacerbations in this setting, linking receptor-dependent infection to an intervention-relevant inflammatory endpoint [46]. This example is especially useful for antibody development because the outcome is not merely viral replication; it is the capacity of receptor-dependent infection to reshape airway inflammation in vivo.

### 4.2. Hepatitis Virus Models: Entry-Factor Humanization and Post-Entry Restrictions

HCV entry-factor models demonstrate that some viruses require coordinated human entry factors rather than a single receptor substitution. Human occludin was shown to be required for HCV infection of mouse cells, and genetically humanized CD81/OCLN systems enabled HCV entry in vivo [47,48]. Subsequent models added innate immune modifications that allowed completion of the HCV life cycle in mice, while minimally humanized CD81 and occludin designs showed how selected human entry determinants can support uptake without replacing every component of the host environment [49,50,51]. This literature is a strong reminder that entry-factor humanization can reveal where the next barrier lies: once entry is solved, replication, innate immunity, or tissue context may become the limiting step.

The HCV field also provides direct evidence for the utility of genetically humanized entry systems in antibody development. Broadly neutralizing antibodies targeting the HCV envelope glycoprotein complex have been characterized in genetically humanized models, and—critically—established infection could be abrogated by antibody treatment in those systems [52,53]. The important lesson is not simply that a mouse can be made permissive, but that the model can connect antibody breadth, authentic entry, viral spread, and in vivo protection. That connection is precisely what cell-culture neutralization assays alone cannot provide.

HBV and HDV models are anchored by NTCP, identified as a functional receptor for both viruses [54]. Mice expressing human NTCP can support HDV entry, and a minimal three-amino-acid modification in mouse NTCP can render mice susceptible to HDV infection in vivo [55,56]. However, hNTCP alone should not be overstated as a complete solution for persistent adult HBV or HDV infection. Comparative NTCP studies, HDV persistence experiments, and NTCP-focused model reviews support a cautious interpretation in which receptor engagement is necessary but not sufficient; hepatocyte state, post-entry compatibility, innate immunity, and liver-humanized context can remain decisive [57,58,59]. This section is therefore a useful counterbalance to receptor-centric thinking: entry is essential, but entry is not the whole disease.

### 4.3. Measles, Poliovirus, Enterovirus, and Arenavirus Models: Tropism, Safety, and Zoonotic Entry

Measles receptor-humanized models illustrate how the choice of receptor determines which disease feature becomes visible. Human CD46-transgenic mice supported studies of measles virus permissiveness and central nervous system infection, whereas hSLAM/CD150 systems better reproduced lymphoid tropism and immunosuppressive features of wild-type measles infection [60,61,62,63,64]. CD46 and SLAM/CD150 models therefore should not be treated as redundant. They answer different questions about viral tropism, immune suppression, and tissue involvement, and they demonstrate why receptor identity must be matched to virus strain and biological endpoint.

Poliovirus PVR/CD155 mice are among the earliest and most conceptually important receptor-humanized viral models. Transgenic expression of the human poliovirus receptor made mice susceptible to poliovirus and enabled studies of poliomyelitis, neurovirulence, and vaccine safety [65,66,67,68,69]. This classical system remains instructive precisely because it demonstrates how a single human entry receptor can transform an otherwise non-permissive species into a platform for neurovirulence and vaccine safety testing. Nevertheless, susceptibility and neurovirulence remain modifiable by interferon signaling and viral sequence determinants, underscoring that even an apparently receptor-defined model must be read in the context of host innate immunity [65,66,67,68,69].

Enterovirus and arenavirus systems further broaden the receptor-humanization framework. SCARB2 was identified as a receptor for EV-A71, and human SCARB2 (hSCARB2) transgenic, hSCARB2/STAT1-deficient, and chimeric mSCARB2/hSCARB2 models have been used to study EV-A71 disease and susceptibility to clinical isolates [70,71,72,73]. These models are directly relevant to vaccine development: an inactivated EV-A71 vaccine produced at bioreactor scale showed protective and cross-protective efficacy in hSCARB2 transgenic mice [74]. Human TfR1 models add a zoonotic perspective. New World hemorrhagic fever arenaviruses use TfR1 in a species-selective manner, and receptor-discovery, receptor-determinant, and human TfR1 mouse studies have linked receptor usage to host range, Junín virus disease mechanisms, and receptor-targeted intervention [75,76,77,78,79,80]. Together, these examples show that receptor humanization can support not only antibody and vaccine efficacy studies but also questions of zoonotic entry, tissue targeting, and receptor-directed therapy.

### 4.4. Comparative Summary of Representative Receptor-Humanized Virus Models

As summarized in Table 1, representative receptor-humanized systems differ not only by viral receptor but also by the type of evidence each model can legitimately support. The comparison intentionally separates entry-factor sufficiency, disease phenotype, antibody testing, vaccine testing, and key limitations so that no model is overinterpreted beyond its validated use. This organization also addresses the major conceptual concern raised by a narrow hACE2-centered review: receptor-humanized mice are not a SARS-CoV-2-specific technology, but a broader experimental strategy spanning respiratory, enteric, hepatotropic, neurotropic, retroviral, and zoonotic viral systems [14,15,16,17,18,19,20,21,22,23,24,25,26,27,28,29,30,31,32,33,34,35,36,37,38,39,40,41,42,43,44,45,46,47,48,49,50,51,52,53,54,55,56,57,58,59,60,61,62,63,64,65,66,67,68,69,70,71,72,73,74,75,76,77,78,79,80].

## 5. Antiviral Antibody Development: Protection, Fc Biology, and Reporting Standards

Receptor-humanized mice are useful for antibody development when the candidate antibody blocks a receptor-dependent entry step, reduces viral spread after entry, or requires an intact tissue environment to evaluate protection. During the SARS-CoV-2 response, humanized-mouse and human antibody studies rapidly identified neutralizing antibody cocktails, structural epitope classes, and cross-neutralizing antibodies that could be moved from in vitro selection into in vivo prioritization [81,82,83,84]. The key contribution of the animal model is not simply to repeat a neutralization assay in a more complex system; it is to determine whether antibody concentration, tissue exposure, viral burden, pathology, and clinical protection align in a living host.

The same logic extends beyond SARS-CoV-2. Potent monoclonal antibodies against MERS-CoV spike showed prophylactic and post-exposure efficacy in hDPP4-related systems [85,86], while HCV broadly neutralizing antibodies were evaluated in genetically humanized entry or life-cycle models [52,53]. These examples show why receptor-humanized models are especially useful at the transition between discovery and development: they can reveal whether an antibody that looks potent in vitro remains protective when tissue access, viral kinetics, and host responses are present. They also help define the limits of antibody breadth, because a model whose susceptibility depends on the relevant human receptor can test whether an antibody blocks the entry event that matters clinically.

These models are also useful for comparing antibody formats. A neutralizing IgG, an Fc-silenced variant, an Fc-enhanced variant, and a cocktail may show similar cell-culture neutralization but differ markedly in tissue protection, inflammatory consequences, or durability in vivo. Receptor-humanized mice allow such comparisons under controlled challenge conditions, provided that the limitations of murine Fc biology are acknowledged. For this reason, Fc engineering should not be treated as a minor formulation detail; it is part of the biological hypothesis being tested, especially when candidate antibodies are intended for prophylaxis in high-risk populations or therapy after infection has already begun [87,88,89,90,91].

**Figure 2:**
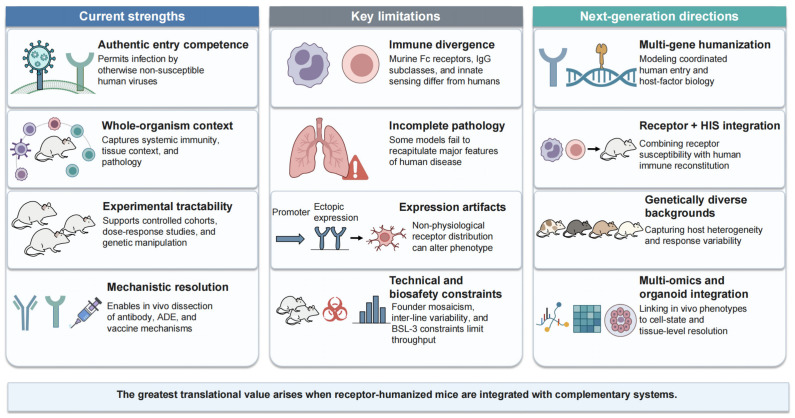
Strengths, limitations, and next-generation directions for receptor-humanized mouse models. Receptor-humanized mouse models provide authentic entry competence for otherwise non-susceptible human viruses and enable mechanistic in vivo studies in a tractable small-animal system. Their major strengths include preservation of whole-organism immune and tissue context, experimental tractability in controlled cohorts, and strong utility for antibody-, ADE-, and vaccine-related mechanistic studies. Their limitations include murine-human immune divergence, incomplete recapitulation of human pathology, receptor expression artifacts caused by non-physiological distribution patterns, and technical as well as biosafety constraints such as founder mosaicism, inter-line variability, and biosafety level 3 (BSL-3) containment requirements. Future advances are likely to arise from multi-gene humanization, integration with humanized immune system (HIS) platforms, expansion into genetically diverse mouse backgrounds, and coupling with multi-omics and organoid-based systems. Taken together, the greatest translational value of receptor-humanized mouse models emerges when they are interpreted within an integrated framework that includes complementary experimental systems.

Fc biology requires particular caution. Receptor-humanized mice usually retain murine Fc receptors and murine effector-cell networks, and these can influence protection or immunopathology in ways that do not map perfectly onto humans. Nevertheless, in vivo studies show that Fc effector functions can be critical for optimal antibody activity against SARS-CoV-2 and HIV-1, while dengue and SARS-CoV studies illustrate why Fc-mediated enhancement or inflammatory skewing must be assessed rather than assumed absent [87,88,89,90,91]. A rigorous antibody workflow should therefore report the receptor-humanized line, receptor expression pattern, challenge virus, antibody isotype, Fc-engineering status, dosing schedule, and the full panel of viral-load, pathology, and immune-cell endpoints. Without those details, a protective effect cannot be cleanly separated from differences in entry window, tissue exposure, or Fc-effector context [81,82,83,84,85,86,87,88,89,90,91]. As outlined in Figure 2, antibody and vaccine studies use overlapping but distinct model features: entry blockade, Fc function, challenge protection, and breadth testing each require specific validation and reporting standards.

## 6. Vaccine Evaluation: Protection, Immune Readouts, and Variant Breadth

For vaccines, receptor-humanized mice test whether immunization prevents authentic receptor-dependent infection and disease. MERS-CoV vaccine candidates have been evaluated in hDPP4 transgenic models, and SARS-CoV-2 mRNA, adenoviral, and intranasal vaccine studies have used hACE2 or related challenge systems to quantify protection in the respiratory tract [92,93,94,95]. These models are most valuable when the vaccine claim depends on preventing infection by a virus whose host range is shaped by a human receptor. They are less informative when the primary endpoint is long-term human immune memory, HLA-restricted T-cell specificity, or mucosal architecture that is not reproduced in the mouse.

Humoral readouts should be paired with tissue and immunological endpoints. Antibody titer alone cannot distinguish durable germinal-center-derived protection from short-lived plasmablast responses, nor can it identify whether protection reflects sterilizing entry blockade, accelerated clearance, or reduced pathology. LNP-based vaccines can induce strong T follicular helper and humoral responses, and SARS-CoV-2 mRNA vaccination can generate persistent germinal-center activity in humans [96,97]. Receptor-humanized challenge models can connect such immune signatures to viral-load and pathology endpoints, but the interpretation remains bounded by murine lymphoid architecture, murine Fc receptors, and the absence of human HLA unless additional humanization is incorporated.

Vaccine safety and interpretation also depend on the type of receptor-humanized model used. A high-susceptibility model may be ideal for detecting incomplete protection because residual infection is easy to measure, but it may overrepresent pathology driven by promoter-defined receptor expression. A more physiological knock-in model may better represent tissue entry but require more sensitive virological or histological endpoints. Neither choice is universally superior. The critical point is to match the model to the vaccine question: prevention of infection, reduction of viral burden, reduction of tissue damage, comparison across variants, or mechanistic analysis of immune correlates [92,93,94,95,96,97,98,99,100,101,102].

T-cell and variant-breadth analyses reinforce the same interpretive boundary. SARS-CoV-specific memory CD8 T cells can protect against lethal challenge in mice, and SARS-CoV-2 epitope mapping in humans has defined immunodominance and immunoprevalence patterns in depth [98,99]. Mouse MHC presentation and human HLA restriction are not interchangeable, however, so T-cell conclusions from receptor-humanized models require either careful framing of their limits or incorporation of HLA/immune-system humanization. Variant benchmarking is similarly powerful but context dependent. Omicron immune escape, vaccine-induced neutralization loss across variants, and mosaic RBD nanoparticle protection against diverse sarbecoviruses show why in vivo breadth testing is needed [100,101,102]. Receptor-humanized models are one important layer of that workflow when the benchmark depends on human receptor usage, but variant studies should use the receptor and immune context most relevant to the intended clinical claim.

## 7. Translational Boundaries: Validation Standards and Model Limits

The most fundamental limitation of receptor-humanized models is precisely what makes them useful: they address the entry step specifically, but entry competence does not automatically reproduce human disease. Human liver chimeric mice demonstrate that organ-humanized platforms may be required for hepatitis virus replication and treatment studies, while human immune-system models are often necessary when vaccine responses, antibody maturation, Fc-effector mechanisms, or immune escape are the primary endpoints [103,104,105,106]. A receptor-humanized mouse should therefore be selected for the question it can answer, not for the disease label attached to the virus.

Receptor expression level, anatomical distribution, and genetic background are the main axes along which interpretation can drift. Overexpression under strong ubiquitous promoters can generate non-physiological tropism, artifactual lethality, or tissue damage that has no counterpart in the natural disease process. Endogenous knock-in configurations typically yield more physiological expression patterns, but often at the cost of a narrower intervention window. Immune background matters as well: mouse and human innate and adaptive responses differ, and host genetic background can reshape pathogenesis even when the virus and receptor are unchanged [6,7,107]. Model descriptions should therefore include receptor locus or promoter, copy number when known, cell-type distribution, age, sex, background strain, viral strain, and the validated endpoint window.

Future model development will likely move from single-receptor substitution toward layered humanization. For hepatitis viruses, liver-humanized mice already show how organ context can complement receptor engineering; for antibody and vaccine studies, immune-system humanization may be needed to evaluate human B-cell maturation, HLA-restricted T-cell responses, and human Fc-effector pathways [103,104,105,106]. At the same time, the field should avoid assuming that more humanization is always better. Each added human component can introduce its own artifacts, engraftment variability, or immunodeficient background. The most useful future systems will therefore be modular: receptor-humanized when the question is entry, organ-humanized when the question is tissue replication, and immune-humanized when the question is adaptive or effector immunity [3,6,103,104,105,106].

A practical validation checklist follows from these limitations. Investigators should ask whether the human receptor is expressed at biologically relevant sites; whether infection is receptor dependent; whether tissue tropism resembles the intended human disease feature; whether viral replication and pathology provide a measurable intervention window; whether antibody or vaccine readouts require human Fc receptors, HLA, or human immune cells; and whether complementary organoid, human cell, immune-humanized, liver-humanized, or non-human primate data are needed for confirmation [1,2,3,4,5,6,7,103,104,105,106,107]. Framed this way, receptor-humanized mice remain powerful but bounded tools: they are neither simple susceptibility switches nor complete human disease replicas.

## 8. Conclusions

Human viral entry receptor mouse models are best understood as a diverse, receptor-defined experimental toolkit extending far beyond hACE2/SARS-CoV-2 systems. hACE2 and hDPP4 models remain central for coronavirus countermeasures, but CD4/CCR5, hCAR, hCD46, hICAM-1, hCD81/hOCLN, hNTCP, hSLAM/CD150, PVR/CD155, hSCARB2, and hTfR1 models demonstrate that the same receptor-humanization principle applies across many medically relevant viral infections [14,15,16,17,18,19,20,21,22,23,24,25,26,27,28,29,30,31,32,33,34,35,36,37,38,39,40,41,42,43,44,45,46,47,48,49,50,51,52,53,54,55,56,57,58,59,60,61,62,63,64,65,66,67,68,69,70,71,72,73,74,75,76,77,78,79,80]. Several limitations of this review and the underlying mouse models should be acknowledged. First, although the scope of this review was deliberately extended beyond coronaviruses, both the available evidence and the depth of discussion remain weighted toward hACE2/SARS-CoV-2 and hDPP4/MERS-CoV models, which are more extensively developed than most non-coronavirus systems. The cross-virus comparisons are therefore qualitative and should not be interpreted as an exhaustive inventory or quantitative ranking. Second, as a narrative review, this article relies on targeted literature selection and does not include a systematic risk-of-bias or certainty-of-evidence assessment; selection and publication bias cannot be excluded. Third, receptor-humanized mice reproduce a defined entry barrier rather than the complete human disease state. Murine immune signaling, Fc receptor and IgG subclass biology, HLA-restricted antigen presentation, stromal and tissue-repair programs, and organ physiology remain incompletely modeled. In addition, promoter choice, transgene copy number, ectopic expression, founder effects, age, sex, genetic background, challenge dose, viral adaptation, and endpoint selection can materially influence apparent efficacy. These limitations should be considered when extrapolating antibody- or vaccine-protection data to humans.

The practical lesson from the available models is that engineering strategy determines interpretation. Random transgenic systems can provide rapid and robust susceptibility, but may introduce promoter- and copy-number-driven artifacts. Endogenous knock-in or minimal receptor-interface editing can better preserve physiological expression, but may produce a narrower disease window and may not remove post-entry species restrictions. Conditional, inducible, and transient expression strategies add flexibility, yet they also require careful controls for timing, tissue distribution, and expression level [8,9,10,11,12,13,18,19,20,21,22,23,24,25,26,27,28,31,32,33,34,35,36,37]. No platform is intrinsically superior to another; each is scientifically defensible only when its construction, receptor localization, infection phenotype, and endpoint window are all aligned with the specific experimental question being asked.

For antiviral antibody and vaccine development, the strongest use of receptor-humanized mice is not to claim comprehensive human disease mimicry, but to test specific entry-dependent hypotheses under controlled in vivo conditions. These models can connect in vitro neutralization to reductions in tissue viral load and pathology, Fc-effector function, and challenge protection, and they are particularly useful when candidate antibodies or vaccines target a human receptor-dependent entry pathway [81,82,83,84,85,86,87,88,89,90,91,92,93,94,95,96,97,98,99,100,101,102,103,104,105,106]. Their translational value therefore depends on restraint as much as breadth: investigators should state exactly which receptor-dependent question the model can answer and identify which human immune or organ-level features require complementary systems.

Future progress will likely come from layered humanization rather than from simply adding more receptor-transgenic lines. Combining receptor-humanized mice with human immune-system reconstitution, organ-humanized platforms, HLA or Fc-receptor humanization, organoids, primary human cell systems, and, when necessary, non-human primate studies will allow entry competence to be interpreted within a more clinically relevant immune and tissue context [1,2,3,4,5,6,7,103,104,105,106,107]. Equally indispensable is transparent reporting: manuscripts should specify the receptor allele design, promoter or locus, copy number where known, tissue distribution, animal background, challenge virus, endpoint window, and—critically—the explicit rationale for selecting that particular model over available alternatives. Used in this disciplined way, human viral entry receptor mouse models can serve as a rigorous bridge between reductionist cell-culture assays and later-stage animal or clinical studies, while avoiding the overinterpretation that has sometimes limited confidence in this field.

## Figures and Tables

**Table 1:** Comparative summary of representative receptor-humanized and entry-competent mouse model platforms discussed in this review.

Virus/Model Group	Humanized Entry Factor(s)	Main Experimental Value	Key Limitation	Representative References
SARS-CoV/SARS-CoV-2	hACE2	Entry-dependent pathogenesis, antibody protection, vaccine and variant challenge	Promoter and tissue distribution can drive non-physiological tropism	[14,15,16,17,18,19,20,21,22,23,24,25,26,27,28]
MERS-CoV	hDPP4/CD26	MERS entry, lung disease and ARDS modeling	Transgenic, knock-in, and mouse-adapted systems are not equivalent	[29,30,31,32,33,34,35,36,37]
HIV-1	hCD4,hCCR5 and hCyclin T1	Entry and early infection constraints	Full pathogenesis often requires human immune-system reconstitution	[38,39,40,41]
Adenovirus vectors	hCAR, hCD46	Vector tropism and antigen-delivery studies	Vector transduction is not the same as natural disease	[42,43,44]
Rhinovirus	hICAM-1	Airway infection and inflammation exacerbation	Primarily models selected major-group rhinovirus questions	[45,46]
HCV	hCD81, hOCLN and additional factors	Entry, viral spread, neutralizing antibody evaluation	Entry humanization may not remove all replication restrictions	[47,48,49,50,51,52,53]
HBV/HDV	hNTCP or minimally humanized NTCP	Receptor engagement and HDV entry	Persistence requires additional hepatocyte and post-entry compatibility	[54,55,56,57,58,59]
Measles virus	hCD46, hSLAM/CD150	Tropism, CNS disease, lymphoid infection and immunosuppression	Receptor choice must match virus strain and disease question	[60,61,62,63,64]
Poliovirus	PVR/CD155	Poliomyelitis, neurovirulence and vaccine safety	Interferon status and viral determinants alter susceptibility	[65,66,67,68,69]
EV-A71/enteroviruses	hSCARB2 or chimeric SCARB2	Disease modeling and vaccine protection	STAT1 deficiency or receptor chimerism changes interpretation	[70,71,72,73,74]
New World arenaviruses	hTfR1	Zoonotic receptor usage and receptor-targeted therapy	Disease phenotype depends on receptor expression and innate immunity	[75,76,77,78,79,80]

Abbreviations: SARS-CoV, severe acute respiratory syndrome coronavirus; SARS-CoV-2, severe acute respiratory syndrome coronavirus 2; hACE2, human angiotensin-converting enzyme 2; MERS-CoV, Middle East respiratory syndrome coronavirus; hDPP4/CD26, human dipeptidyl peptidase 4/CD26; ARDS, acute respiratory distress syndrome; HIV-1, human immunodeficiency virus type 1; hCD4, human cluster of differentiation 4; hCCR5, human C-C chemokine receptor type 5; hCyclin T1, human cyclin T1; hCAR, human coxsackievirus and adenovirus receptor; hCD46, human CD46; hICAM-1, human intercellular adhesion molecule 1; HCV, hepatitis C virus; hCD81, human cluster of differentiation 81; hOCLN, human occludin; HBV, hepatitis B virus; HDV, hepatitis D virus; hNTCP, human sodium taurocholate cotransporting polypeptide; hSLAM/CD150, human signaling lymphocytic activation molecule/CD150; CNS, central nervous system; PVR/CD155, poliovirus receptor/CD155; EV-A71, enterovirus A71; hSCARB2, human scavenger receptor class B member 2; STAT1, signal transducer and activator of transcription 1; hTfR1, human transferrin receptor 1.

## Data Availability

No new data were created or analyzed in this study. Data sharing is not applicable to this article.

## References

[1] Masemann D., Ludwig S., Planz O. (2020). Advances in transgenic mouse models to study infections by human pathogenic viruses. Int. J. Mol. Sci..

[2] Muñoz-Fontela C., Dowling W.E., Funnell S.G.P., Gsell P.S., Riveros-Balta A.X., Albrecht R.A., Andersen H., Baric R.S., Carroll M.W., Cavaleri M. (2020). Animal models for COVID-19. Nature.

[3] Wahl A., Garcia J.V. (2025). Humanized mouse systems to study viral infection: A new era in immunology research. Annu. Rev. Immunol..

[4] Douam F., Ploss A. (2018). The use of humanized mice for studies of viral pathogenesis and immunity. Curr. Opin. Virol..

[5] Lai F., Chen Q. (2018). Humanized mouse models for the study of infection and pathogenesis. Viruses.

[6] Shultz L.D., Brehm M.A., Garcia-Martinez J.V., Greiner D.L. (2012). Humanized mice for immune system investigation: Progress, promise and challenges. Nat. Rev. Immunol..

[7] Mestas J., Hughes C.C.W. (2004). Of mice and not men: Differences between mouse and human immunology. J. Immunol..

[8] Gurumurthy C.B., Lloyd K.C.K. (2019). Generating mouse models for biomedical research: Technological advances. Dis. Model. Mech..

[9] Gordon J.W., Ruddle F.H. (1981). Integration and stable germ line transmission of genes injected into mouse pronuclei. Science.

[10] Palmiter R.D., Brinster R.L. (1986). Germ-line transformation of mice. Annu. Rev. Genet..

[11] Gossen M., Bujard H. (1992). Tight control of gene expression in mammalian cells by tetracycline-responsive promoters. Proc. Natl. Acad. Sci. USA.

[12] Feil R., Brocard J., Mascrez B., LeMeur M., Metzger D., Chambon P. (1996). Ligand-activated site-specific recombination in mice. Proc. Natl. Acad. Sci. USA.

[13] Anzalone A.V., Randolph P.B., Davis J.R., Sousa A.A., Koblan L.W., Levy J.M., Chen P.J., Wilson C., Newby G.A., Raguram A. (2019). Search-and-replace genome editing without double-strand breaks or donor DNA. Nature.

[14] Li F., Li W., Farzan M., Harrison S.C. (2005). Structure of SARS coronavirus spike receptor-binding domain complexed with receptor. Science.

[15] Hoffmann M., Kleine-Weber H., Schroeder S., Krüger N., Herrler T., Erichsen S., Schiergens T.S., Herrler G., Wu N.-H., Nitsche A. (2020). SARS-CoV-2 cell entry depends on ACE2 and TMPRSS2 and is blocked by a clinically proven protease inhibitor. Cell.

[16] Lan J., Ge J., Yu J., Shan S., Zhou H., Fan S., Zhang Q., Shi X., Wang Q., Zhang L. (2020). Structure of the SARS-CoV-2 spike receptor-binding domain bound to the ACE2 receptor. Nature.

[17] Shang J., Ye G., Shi K., Wan Y., Luo C., Aihara H., Geng Q., Auerbach A., Li F. (2020). Structural basis of receptor recognition by SARS-CoV-2. Nature.

[18] McCray P.B., Pewe L., Wohlford-Lenane C., Hickey M., Manzel L., Shi L., Netland J., Jia H.P., Halabi C., Sigmund C.D. (2007). Lethal infection of K18-hACE2 mice infected with severe acute respiratory syndrome coronavirus. J. Virol..

[19] Bao L., Deng W., Huang B., Gao H., Liu J., Ren L., Wei Q., Yu P., Xu Y., Qi F. (2020). The pathogenicity of SARS-CoV-2 in hACE2 transgenic mice. Nature.

[20] Jiang R.-D., Liu M.-Q., Chen Y., Shan C., Zhou Y.-W., Shen X.-R., Li Q., Zhang L., Zhu Y., Si H.-R. (2020). Pathogenesis of SARS-CoV-2 in transgenic mice expressing human angiotensin-converting enzyme 2. Cell.

[21] Winkler E.S., Bailey A.L., Kafai N.M., Nair S., McCune B.T., Yu J., Fox J.M., Chen R.E., Earnest J.T., Keeler S.P. (2020). SARS-CoV-2 infection of human ACE2-transgenic mice causes severe lung inflammation and impaired function. Nat. Immunol..

[22] Oladunni F.S., Park J.-G., Pino P.A., Gonzalez O., Akhter A., Allué-Guardia A., Olmo-Fontánez A., Gautam S., Garcia-Vilanova A., Ye C. (2020). Lethality of SARS-CoV-2 infection in K18 human angiotensin-converting enzyme 2 transgenic mice. Nat. Commun..

[23] Sun S.-H., Chen Q., Gu H.-J., Yang G., Wang Y.-X., Huang X.-Y., Liu S.-S., Zhang N.-N., Li X.-F., Xiong R. (2020). A mouse model of SARS-CoV-2 infection and pathogenesis. Cell Host Microbe.

[24] Zhou X., Sun W., Zhang Y., Gu H., Wang R., Xie P., Zhu Y., Qiu M., Ding X., Wang H. (2023). A novel hACE2 knock-in mouse model recapitulates pulmonary and intestinal SARS-CoV-2 infection. Front. Microbiol..

[25] Song I.W., Washington M., Leynes C., Hsu J., Rayavara K., Bae Y., Haelterman N., Chen Y., Jiang M.M., Drelich A. (2024). Generation of a humanized mAce2 and a conditional hACE2 mouse models permissive to SARS-CoV-2 infection. Mamm. Genome.

[26] Liu G., Zhang M., Wu B., Zhang C., Wang Y., Han X., Wang R., Li L., Wei Y., Sun Y. (2024). A highly susceptible hACE2-transgenic mouse model for SARS-CoV-2 research. Front. Microbiol..

[27] Israelow B., Song E., Mao T., Lu P., Meir A., Liu F., Alfajaro M.M., Wei J., Dong H., Homer R.J. (2020). Mouse model of SARS-CoV-2 reveals inflammatory role of type I interferon signaling. J. Exp. Med..

[28] Dinnon K.H., Leist S.R., Schäfer A., Edwards C.E., Martinez D.R., Montgomery S.A., West A., Yount B.L., Hou Y.J., Adams L.E. (2020). A mouse-adapted model of SARS-CoV-2 to test COVID-19 countermeasures. Nature.

[29] Raj V.S., Mou H., Smits S.L., Dekkers D.H.W., Müller M.A., Dijkman R., Muth D., Demmers J.A.A., Zaki A., Fouchier R.A.M. (2013). Dipeptidyl peptidase 4 is a functional receptor for the emerging human coronavirus-EMC. Nature.

[30] Cockrell A.S., Peck K.M., Yount B.L., Agnihothram S.S., Scobey T., Curnes N.R., Baric R.S., Heise M.T. (2014). Mouse dipeptidyl peptidase 4 is not a functional receptor for Middle East respiratory syndrome coronavirus infection. J. Virol..

[31] Zhao J., Li K., Wohlford-Lenane C., Agnihothram S.S., Fett C., Zhao J., Gale M.J., Baric R.S., Enjuanes L., Gallagher T. (2014). Rapid generation of a mouse model for Middle East respiratory syndrome. Proc. Natl. Acad. Sci. USA.

[32] Agrawal A.S., Garron T., Tao X., Peng B.-H., Wakamiya M., Chan T.-S., Couch R.B., Tseng C.-T.K. (2015). Generation of a transgenic mouse model of Middle East respiratory syndrome coronavirus infection and disease. J. Virol..

[33] Zhao G., Jiang Y., Qiu H., Gao T., Zeng Y., Guo Y., Yu H., Li J., Kou Z., Du L. (2015). Multi-organ damage in human dipeptidyl peptidase 4 transgenic mice infected with Middle East respiratory syndrome-coronavirus. PLoS ONE.

[34] Li K., Wohlford-Lenane C., Perlman S., Zhao J., Jewell A.K., Reznikov L.R., Gibson-Corley K.N., Meyerholz D.K., McCray P.B. (2016). Middle East respiratory syndrome coronavirus causes multiple organ damage and lethal disease in mice transgenic for human dipeptidyl peptidase 4. J. Infect. Dis..

[35] Cockrell A.S., Yount B.L., Scobey T., Jensen K., Douglas M., Beall A., Tang X.C., Marasco W.A., Heise M.T., Baric R.S. (2016). A mouse model for MERS coronavirus-induced acute respiratory distress syndrome. Nat. Microbiol..

[36] Li K., Wohlford-Lenane C.L., Channappanavar R., Park J.-E., Earnest J.T., Bair T.B., Bates A.M., Brogden K.A., Flaherty H.A., Gallagher T. (2017). Mouse-adapted MERS coronavirus causes lethal lung disease in human DPP4 knockin mice. Proc. Natl. Acad. Sci. USA.

[37] Fan C., Wu X., Liu Q., Li Q., Liu S., Lu J., Yang Y., Cao Y., Huang W., Liang C. (2018). A human DPP4-knockin mouse’s susceptibility to infection by authentic and pseudotyped MERS-CoV. Viruses.

[38] Browning J., Horner J.W., Pettoello-Mantovani M., Raker C., Yurasov S., DePinho R.A., Goldstein H. (1997). Mice transgenic for human CD4 and CCR5 are susceptible to HIV infection. Proc. Natl. Acad. Sci. USA.

[39] Seay K., Qi X., Zheng J.H., Zhang C., Chen K., Dutta M., Deneroff K., Ochsenbauer C., Kappes J.C., Littman D.R. (2013). Mice transgenic for CD4-specific human CD4, CCR5 and cyclin T1 expression: A new model for investigating HIV-1 transmission and treatment efficacy. PLoS ONE.

[40] Garcia J.V. (2016). Humanized mice for HIV and AIDS research. Curr. Opin. Virol..

[41] Gillgrass A., Wessels J.M., Yang J.X., Kaushic C. (2021). Advances in humanized mouse models to improve understanding of HIV-1 pathogenesis and immune responses. Front. Immunol..

[42] Wan Y.Y., Leon R.P., Marks R., Cham C.M., Schaack J., Gajewski T.F., DeGregori J. (2000). Transgenic expression of the coxsackie/adenovirus receptor enables adenoviral-mediated gene delivery in naïve T cells. Proc. Natl. Acad. Sci. USA.

[43] Zhang Y., Bergelson J.M. (2005). Adenovirus receptors. J. Virol..

[44] Verhaagh S., de Jong E., Goudsmit J., Lecollinet S., Gillissen G., de Swart R.L., Rimmelzwaan G.F., Osterhaus A.D.M.E. (2006). Human CD46-transgenic mice in studies involving replication-incompetent adenoviral type 35 vectors. J. Gen. Virol..

[45] Bartlett N.W., Walton R.P., Edwards M.R., Aniscenko J., Caramori G., Zhu J., Glanville N., Choy K.J., Jourdan P., Burnet J. (2008). Mouse models of rhinovirus-induced disease and exacerbation of allergic airway inflammation. Nat. Med..

[46] Traub S., Nikonova A., Carruthers A., Dunmore R., Vousden K.A., Gogsadze L., Hao W., Zhu Q., Bernard K., Zhu J. (2013). An anti-human ICAM-1 antibody inhibits rhinovirus-induced exacerbations of lung inflammation. PLoS Pathog..

[47] Ploss A., Evans M.J., Gaysinskaya V.A., Panis M., You H., de Jong Y.P., Rice C.M. (2009). Human occludin is a hepatitis C virus entry factor required for infection of mouse cells. Nature.

[48] Dorner M., Horwitz J.A., Robbins J.B., Barry W.T., Feng Q., Mu K., Jones C.T., Schoggins J.W., Catanese M.T., Burton D.R. (2011). A genetically humanized mouse model for hepatitis C virus infection. Nature.

[49] Dorner M., Horwitz J.A., Donovan B.M., Labitt R.N., Budell W.C., Friling T., Vogt A., Catanese M.T., Satoh T., Kawai T. (2013). Completion of the entire hepatitis C virus life cycle in genetically humanized mice. Nature.

[50] Dorner M., Rice C.M., Ploss A. (2013). Study of hepatitis C virus entry in genetically humanized mice. Methods.

[51] Ding Q., von Schaewen M., Hrebikova G., Heller B., Sandmann L., Plaas M., Ploss A. (2017). Mice expressing minimally humanized CD81 and occludin genes support hepatitis C virus uptake in vivo. J. Virol..

[52] Giang E., Dorner M., Prentoe J.C., Dreux M., Evans M.J., Bukh J., Rice C.M., Ploss A., Burton D.R., Law M. (2012). Human broadly neutralizing antibodies to the envelope glycoprotein complex of hepatitis C virus. Proc. Natl. Acad. Sci. USA.

[53] de Jong Y.P., Dorner M., Mommersteeg M.C., Xiao J.W., Balazs A.B., Robbins J.B., Winer B.Y., Gerges S., Vega K., Labitt R.N. (2014). Broadly neutralizing antibodies abrogate established hepatitis C virus infection. Sci. Transl. Med..

[54] Yan H., Zhong G., Xu G., He W., Jing Z., Gao Z., Huang Y., Qi Y., Peng B., Wang H. (2012). Sodium taurocholate cotransporting polypeptide is a functional receptor for human hepatitis B and D virus. eLife.

[55] He W., Ren B., Mao F., Jing Z., Li Y., Liu Y., Peng B., Yan H., Qi Y., Sun Y. (2015). Hepatitis D virus infection of mice expressing human sodium taurocholate co-transporting polypeptide. PLoS Pathog..

[56] He W., Cao Z., Mao F., Ren B., Li Y., Li D., Li H., Peng B., Yan H., Qi Y. (2016). Modification of three amino acids in sodium taurocholate cotransporting polypeptide renders mice susceptible to infection with hepatitis D virus in vivo. J. Virol..

[57] Lempp F.A., Wiedtke E., Qu B., Roques P., Chemin I., Vondran F.W.R., Le Grand R., Grimm D., Urban S. (2017). Sodium taurocholate cotransporting polypeptide is the limiting host factor of hepatitis B virus infection in macaque and pig hepatocytes. Hepatology.

[58] Giersch K., Hermanussen L., Volz T., Kah J., Allweiss L., Casey J., Sureau C., Dandri M., Lütgehetmann M. (2021). Murine hepatocytes do not support persistence of hepatitis D virus mono-infection in vivo. Liver Int..

[59] Wettengel J.M., Burwitz B.J. (2020). Innovative HBV animal models based on the entry receptor NTCP. Viruses.

[60] Horvat B., Rivailler P., Varior-Krishnan G., Cardoso A., Gerlier D., Rabourdin-Combe C. (1996). Transgenic mice expressing human measles virus receptor CD46 provide cells exhibiting different permissivities to measles virus infection. J. Virol..

[61] Rall G.F., Manchester M., Daniels L.R., Callahan E.M., Belman A.R., Oldstone M.B.A. (1997). A transgenic mouse model for measles virus infection of the brain. Proc. Natl. Acad. Sci. USA.

[62] Oldstone M.B.A., Lewicki H., Thomas D., Tishon A., Dales S., Patterson J., Manchester M., Homann D., Naniche D., Holz A. (1999). Measles virus infection in a transgenic model: Virus-induced immunosuppression and central nervous system disease. Cell.

[63] Sellin C.I., Davoust N., Guillaume V., Baas D., Buckland R., Wild T.F., Horvat B. (2006). High pathogenicity of wild-type measles virus infection in CD150 (SLAM) transgenic mice. J. Virol..

[64] Ohno S., Ono N., Takeda M., Takeuchi K., Yanagi Y. (2007). Measles virus infection of SLAM (CD150) knockin mice reproduces tropism and immunosuppression. J. Virol..

[65] Ren R., Costantini F., Gorgacz E.J., Lee J.J., Racaniello V.R. (1990). Transgenic mice expressing a human poliovirus receptor: A new model for poliomyelitis. Cell.

[66] Koike S., Taya C., Kurata T., Abe S., Ise I., Yonekawa H., Nomoto A. (1991). Transgenic mice susceptible to poliovirus. Proc. Natl. Acad. Sci. USA.

[67] Horie H., Koike S., Kurata T., Sato-Yoshida Y., Ise I., Ota Y., Abe S., Hioki K., Kato H., Taya C. (1994). Transgenic mice carrying the human poliovirus receptor: New animal model for study of poliovirus neurovirulence. J. Virol..

[68] Abe S., Ota Y., Koike S., Kurata T., Horie H., Nomura T., Hashizume S., Nomoto A. (1995). Neurovirulence test for oral live poliovaccines using poliovirus-sensitive transgenic mice. Virology.

[69] Ohka S., Igarashi H., Nagata N., Sakai M., Koike S., Nochi T., Kiyono H., Nomoto A. (2007). Establishment of a poliovirus oral infection system in human poliovirus receptor-expressing transgenic mice that are deficient in alpha/beta interferon receptor. J. Virol..

[70] Yamayoshi S., Yamashita Y., Li J., Hanagata N., Minowa T., Takemura T., Koike S. (2009). Scavenger receptor B2 is a cellular receptor for enterovirus 71. Nat. Med..

[71] Lin Y.-W., Yu S.-L., Shao H.-Y., Lin H.-Y., Liu C.-C., Hsiao K.-N., Chitra E., Tsou Y.-L., Chang H.-W., Sia C. (2013). Human SCARB2 transgenic mice as an infectious animal model for enterovirus 71. PLoS ONE.

[72] Liou A.-T., Wu S.-Y., Liao C.-C., Chang Y.-S., Chang C.-S., Shih C. (2016). A new animal model containing human SCARB2 and lacking stat-1 is highly susceptible to EV71. Sci. Rep..

[73] Yang C.-H., Liang C.-T., Jiang S.-T., Chen K.-H., Yang C.-C., Cheng M.-L., Ho H.-Y., Jong Y.-J., Lin T.-Y., Chiu C.-H. (2019). A novel murine model expressing a chimeric mSCARB2/hSCARB2 receptor is highly susceptible to oral infection with clinical isolates of enterovirus 71. J. Virol..

[74] Wu C.-Y., Lin Y.-W., Kuo C.-H., Liu W.-H., Tai H.-F., Pan C.-H., Chen Y.-T., Hsiao P.-W., Chan C.-H., Chang C.-C. (2015). Inactivated enterovirus 71 vaccine produced by 200-L scale serum-free microcarrier bioreactor system provides cross-protective efficacy in human SCARB2 transgenic mouse. PLoS ONE.

[75] Radoshitzky S.R., Abraham J., Spiropoulou C.F., Kuhn J.H., Nguyen D., Li W., Nagel J., Schmidt P.J., Nunberg J.H., Andrews N.C. (2007). Transferrin receptor 1 is a cellular receptor for New World haemorrhagic fever arenaviruses. Nature.

[76] Radoshitzky S.R., Kuhn J.H., Spiropoulou C.F., Albariño C.G., Nguyen D.P., Salazar-Bravo J., Dorfman T., Lee A.S., Wang E., Ross S.R. (2008). Receptor determinants of zoonotic transmission of New World hemorrhagic fever arenaviruses. Proc. Natl. Acad. Sci. USA.

[77] Abraham J., Kwong J.A., Albariño C.G., Lu J.G., Radoshitzky S.R., Salazar-Bravo J., Farzan M., Spiropoulou C.F., Choe H. (2009). Host-species transferrin receptor 1 orthologs are cellular receptors for nonpathogenic New World clade B arenaviruses. PLoS Pathog..

[78] Hickerson B.T., Sefing E.J., Bailey K.W., Van Wettere A.J., Penichet M.L., Gowen B.B. (2020). Type I interferon underlies severe disease associated with Junín virus infection in mice. eLife.

[79] Hickerson B.T., Daniels-Wells T.R., Payes C., Clark L.E., Candelaria P.V., Bailey K.W., Sefing E.J., Zink S., Ziegenbein J., Abraham J. (2022). Host receptor-targeted therapeutic approach to counter pathogenic New World mammarenavirus infections. Nat. Commun..

[80] Iyer K., Zeltina A., Canard B., Gutsche I. (2024). Entry inhibitors as arenavirus antivirals. Front. Microbiol..

[81] Hansen J., Baum A., Pascal K.E., Russo V., Giordano S., Wloga E., Fulton B.O., Yan Y., Koon K., Patel K. (2020). Studies in humanized mice and convalescent humans yield a SARS-CoV-2 antibody cocktail. Science.

[82] Baum A., Fulton B.O., Wloga E., Copin R., Pascal K.E., Russo V., Giordano S., Lanza K., Negron N., Ni M. (2020). Antibody cocktail to SARS-CoV-2 spike protein prevents rapid mutational escape seen with individual antibodies. Science.

[83] Barnes C.O., Jette C.A., Abernathy M.E., Dam K.-M.A., Esswein S.R., Gristick H.B., Malyutin A.G., Sharaf N.G., Huey-Tubman K.E., Lee Y.E. (2020). SARS-CoV-2 neutralizing antibody structures inform therapeutic strategies. Nature.

[84] Pinto D., Park Y.-J., Beltramello M., Walls A.C., Tortorici M.A., Bianchi S., Jaconi S., Culap K., Zatta F., De Marco A. (2020). Cross-neutralization of SARS-CoV-2 by a human monoclonal SARS-CoV antibody. Nature.

[85] Corti D., Zhao J., Pedotti M., Simonelli L., Agnihothram S., Fett C., Fernandez-Rodriguez B., Foglierini M., Agatic G., Vanzetta F. (2015). Prophylactic and postexposure efficacy of a potent human monoclonal antibody against MERS coronavirus. Proc. Natl. Acad. Sci. USA.

[86] Pascal K.E., Coleman C.M., Mujica A.O., Kamat V., Badithe A., Fairhurst J., Hunt C., Strein J., Berrebi A., Sisk J.M. (2015). Pre- and postexposure efficacy of fully human antibodies against Spike protein in a novel humanized mouse model of MERS-CoV infection. Proc. Natl. Acad. Sci. USA.

[87] Winkler E.S., Gilchuk P., Yu J., Bailey A.L., Chen R.E., Chong Z., Zost S.J., Jang H., Huang Y., Allen J.D. (2021). Human neutralizing antibodies against SARS-CoV-2 require intact Fc effector functions for optimal therapeutic protection. Cell.

[88] Bournazos S., Klein F., Pietzsch J., Seaman M.S., Nussenzweig M.C., Ravetch J.V. (2014). Broadly neutralizing anti-HIV-1 antibodies require Fc effector functions for in vivo activity. Cell.

[89] Balsitis S.J., Williams K.L., Lachica R., Flores D., Kyle J.L., Mehlhop E., Johnson S., Diamond M.S., Beatty P.R., Harris E. (2010). Lethal antibody enhancement of dengue disease in mice is prevented by Fc modification. PLoS Pathog..

[90] Liu L., Wei Q., Lin Q., Fang J., Wang H., Kwok H., Tang H., Nishiura K., Peng J., Tan Z. (2019). Anti-spike IgG causes severe acute lung injury by skewing macrophage responses during acute SARS-CoV infection. JCI Insight.

[91] Nimmerjahn F., Ravetch J.V. (2008). Fcγ receptors as regulators of immune responses. Nat. Rev. Immunol..

[92] Wang L., Shi W., Joyce M.G., Modjarrad K., Zhang Y., Leung K., Lees C.R., Zhou T., Yassine H.M., Kanekiyo M. (2015). Evaluation of candidate vaccine approaches for MERS-CoV. Nat. Commun..

[93] Hashem A.M., Algaissi A., Agrawal A.S., Al-Amri S.S., Alhabbab R.Y., Sohrab S.S., Almasoud A.S., Alharbi N.K., Peng B.H., Russell M. (2019). A highly immunogenic, protective, and safe adenovirus-based vaccine expressing Middle East respiratory syndrome coronavirus S1-CD40L fusion protein in a transgenic human dipeptidyl peptidase 4 mouse model. J. Infect. Dis..

[94] Corbett K.S., Edwards D.K., Leist S.R., Abiona O.M., Boyoglu-Barnum S., Gillespie R.A., Himansu S., Schäfer A., Ziwawo C.T., DiPiazza A.T. (2020). SARS-CoV-2 mRNA vaccine design enabled by prototype pathogen preparedness. Nature.

[95] Hassan A.O., Kafai N.M., Dmitriev I.P., Fox J.M., Smith B.K., Harvey I.B., Chen R.E., Winkler E.S., Wessel A.W., Case J.B. (2020). A single-dose intranasal ChAd vaccine protects upper and lower respiratory tracts against SARS-CoV-2. Cell.

[96] Alameh M.G., Tombácz I., Bettini E., Lederer K., Sittplangkoon C., Wilmore J.R., Gaudette B.T., Soliman O.Y., Pine M., Hicks P. (2021). Lipid nanoparticles enhance the efficacy of mRNA and protein subunit vaccines by inducing robust T follicular helper cell and humoral responses. Immunity.

[97] Turner J.S., O’Halloran J.A., Kalaidina E., Kim W., Schmitz A.J., Zhou J.Q., Lei T., Thapa M., Chen R.E., Case J.B. (2021). SARS-CoV-2 mRNA vaccines induce persistent human germinal centre responses. Nature.

[98] Channappanavar R., Fett C., Zhao J., Meyerholz D.K., Perlman S. (2014). Virus-specific memory CD8 T cells provide substantial protection from lethal severe acute respiratory syndrome coronavirus infection. J. Virol..

[99] Tarke A., Sidney J., Kidd C.K., Dan J.M., Ramirez S.I., Yu E.D., Mateus J., da Silva Antunes R., Moore E., Rubiro P. (2021). Comprehensive analysis of T cell immunodominance and immunoprevalence of SARS-CoV-2 epitopes in COVID-19 cases. Cell Rep. Med..

[100] Cele S., Jackson L., Khoury D.S., Khan K., Moyo-Gwete T., Tegally H., San J.E., Cromer D., Scheepers C., Amoako D.G. (2022). Omicron extensively but incompletely escapes Pfizer BNT162b2 neutralization. Nature.

[101] Garcia-Beltran W.F., Lam E.C., Denis K.S., Nitido A.D., Garcia Z.H., Hauser B.M., Feldman J., Pavlovic M.N., Gregory D.J., Poznansky M.C. (2021). Multiple SARS-CoV-2 variants escape neutralization by vaccine-induced humoral immunity. Cell.

[102] Cohen A.A., van Doremalen N., Greaney A.J., Andersen H., Sharma A., Starr T.N., Keeffe J.R., Fan C., Schulz J.E., Gnanapragasam P.N.P. (2022). Mosaic RBD nanoparticles protect against challenge by diverse sarbecoviruses in animal models. Science.

[103] Bissig K.-D., Le T.T., Woods N.-B., Verma I.M. (2007). Repopulation of adult and neonatal mice with human hepatocytes: A chimeric animal model. Proc. Natl. Acad. Sci. USA.

[104] Mercer D.F., Schiller D.E., Elliott J.F., Douglas D.N., Hao C., Rinfret A., Addison W.R., Fischer K.P., Churchill T.A., Lakey J.R. (2001). Hepatitis C virus replication in mice with chimeric human livers. Nat. Med..

[105] Bissig K.-D., Wieland S.F., Tran P., Isogawa M., Le T.T., Chisari F.V., Verma I.M. (2010). Human liver chimeric mice provide a model for hepatitis B and C virus infection and treatment. J. Clin. Investig..

[106] Douam F., Ziegler C.G.K., Hrebikova G., Fant B., Leach R., Parsons L., Wang W., Gaska J.M., Winer B.Y., Heller B. (2018). Selective expansion of myeloid and natural killer cells in humanized mice yields human-like vaccine responses. Nat. Commun..

[107] Ferris M.T., Aylor D.L., Bottomly D., Whitmore A.C., Aicher L.D., Bell T.A., Bradel-Tretheway B., Bryan J.T., Buus R.J., Gralinski L.E. (2013). Modeling host genetic regulation of influenza pathogenesis in the Collaborative Cross. PLoS Pathog..

